# Proactive Electronic Visits for Smoking Cessation and Chronic Obstructive Pulmonary Disease Screening in Primary Care: Randomized Controlled Trial of Feasibility, Acceptability, and Efficacy

**DOI:** 10.2196/38663

**Published:** 2022-08-30

**Authors:** Jennifer Dahne, Marty S Player, Charlie Strange, Matthew J Carpenter, Dee W Ford, Kathryn King, Sarah Miller, Ryan Kruis, Elizabeth Hawes, Johanna E Hidalgo, Vanessa A Diaz

**Affiliations:** 1 Department of Psychiatry and Behavioral Sciences Medical University of South Carolina Charleston, SC United States; 2 Hollings Cancer Center Medical University of South Carolina Charleston, SC United States; 3 Department of Family Medicine Medical University of South Carolina Charleston, SC United States; 4 Division of Pulmonary and Critical Care Medicine Medical University of South Carolina Charleston, SC United States; 5 Department of Public Health Sciences Medical University of South Carolina Charleston, SC United States; 6 Department of Pediatrics Medical University of South Carolina Charleston, SC United States; 7 College of Nursing Medical University of South Carolina Charleston, SC United States; 8 Center for Telehealth Medical University of South Carolina Charleston, SC United States

**Keywords:** electronic visits, e-visit, COPD, chronic obstructive pulmonary disease, smoking cessation, telehealth, electronic health record, patient portal, EHR, feasibility, efficacy, intervention, screening, diagnosis, prevention, treatment, management, acceptability, pulmonary, function

## Abstract

**Background:**

Most smokers with chronic obstructive pulmonary disease (COPD) have not yet been diagnosed, a statistic that has remained unchanged for over two decades. A dual-focused telehealth intervention that promotes smoking cessation, while also facilitating COPD screening, could help address national priorities to improve the diagnosis, prevention, treatment, and management of COPD. The purpose of this study was to preliminarily evaluate an integrated asynchronous smoking cessation and COPD screening e-visit (electronic visit) that could be delivered proactively to adult smokers at risk for COPD, who are treated within primary care.

**Objective:**

The aims of this study were (1) to examine e-visit feasibility and acceptability, particularly as compared to in-lab diagnostic pulmonary function testing (PFT), and (2) to examine the efficacy of smoking cessation e-visits relative to treatment as usual (TAU), all within primary care.

**Methods:**

In a randomized clinical trial, 125 primary care patients who smoke were randomized 2:1 to receive either proactive e-visits or TAU. Participants randomized to the e-visit condition were screened for COPD symptoms via the COPD Assessment in Primary Care to Identify Undiagnosed Respiratory Disease and Exacerbation Risk (CAPTURE). Those with scores ≥2 were invited to complete both home spirometry and in-lab PFTs, in addition to two smoking cessation e-visits. Smoking cessation e-visits assessed smoking history and motivation to quit and included completion of an algorithm to determine the best Food and Drug Administration–approved cessation medication to prescribe. Primary outcomes included measures related to (1) e-visit acceptability, feasibility, and treatment metrics; (2) smoking cessation outcomes (cessation medication use, 24-hour quit attempts, smoking reduction ≥50%, self-reported abstinence, and biochemically confirmed abstinence); and (3) COPD screening outcomes.

**Results:**

Of 85 participants assigned to the e-visits, 64 (75.3%) were invited to complete home spirometry and in-lab PFTs based on CAPTURE. Among those eligible for spirometry, 76.6% (49/64) completed home spirometry, and 35.9% (23/64) completed in-lab PFTs. At 1 month, all cessation outcomes favored the e-visit, with a significant effect for cessation medication use (odds ratio [OR]=3.22). At 3 months, all cessation outcomes except for 24-hour quit attempts favored the e-visit, with significant effects for cessation medication use (OR=3.96) and smoking reduction (OR=3.09).

**Conclusions:**

A proactive, asynchronous e-visit for smoking cessation and COPD screening may offer a feasible, efficacious approach for broad interventions within primary care.

**Trial Registration:**

ClinicalTrials.gov NCT04155073; https://clinicaltrials.gov/ct2/show/NCT04155073

## Introduction

Cigarette smoking remains the leading cause of preventable death globally and is responsible for more than 480,000 deaths each year in the United States [1]. A total of 21% percent of tobacco-related deaths are caused by chronic obstructive pulmonary disease (COPD), a progressive inflammatory lung disease that causes airflow obstruction and breathing-related problems [1,2]. Between 45% and 72% of smokers with COPD have not yet been diagnosed [3,4], a statistic that has remained largely unchanged for over two decades [4]. As noted in a recent viewpoint article by Yawn and Martinez [5], “COPD screening must develop better, more symptom-based tools and appropriate follow-up support.” A dual-focused intervention that simultaneously promotes smoking cessation, while also facilitating COPD screening, could address national priorities to improve the diagnosis, prevention, treatment, and management of COPD [6].

The vast majority (~70%) of adult smokers visit a primary care provider at least once per year, making primary care an ideal environment within which to identify smokers, provide evidence-based smoking cessation treatment, and screen for COPD [7-9]. Within the primary care setting, prior studies demonstrate up to a fourfold increase in COPD diagnosis when using screening tools to identify respiratory symptoms [10-12]. Although not COPD-specific, our team previously developed an asynchronous e-visit (electronic visit) to be delivered within the primary care environment to patients identified as smokers via the electronic health record (EHR) [13]. This e-visit was developed based on best practice guidelines [7] for smoking cessation treatment within primary care—the 5 A’s (ask, advise, assess, assist, and arrange). Results from an initial evaluation of the asynchronous smoking cessation e-visit as compared to treatment as usual (TAU) within primary care indicated high feasibility and acceptability with cessation outcomes that favored the e-visit condition at both 1 (odds ratios [ORs] 2.10-5.39) and 3 months (ORs 1.31-4.67) [13].

The purpose of this study was to preliminarily evaluate an integrated asynchronous smoking cessation and COPD screening e-visit that could be delivered proactively to adult smokers at risk for COPD, treated within primary care. Prior studies have evaluated the feasibility, acceptability, and validity of remote home spirometry and have found high test-retest reliability when compared to in-clinic assessments [14,15], high adherence rates [16], and high patient satisfaction [15,16]. As such, we opted to leverage our existing asynchronous smoking cessation e-visit platform and add to it remote, telehealth-facilitated COPD screening and completion of remote home spirometry for those eligible. The aims of this study were (1) to examine e-visit (for smoking cessation and remote home spirometry) feasibility and acceptability, particularly as compared to in-lab diagnostic pulmonary function testing (PFTs), and (2) to examine the efficacy of smoking cessation e-visits relative to TAU, all within primary care.

## Methods

### Ethics Approval

All study procedures were approved by the Medical University of South Carolina (MUSC) institutional review board (PRO00086016), and the trial was preregistered with ClinicalTrials.gov (NCT04155073). Eligible patients were scheduled to complete informed consent remotely with a member of the study team. Consent was completed either electronically or via mail, in both cases paired with a discussion with a member of the research team.

### Participants

**Figure 1:**
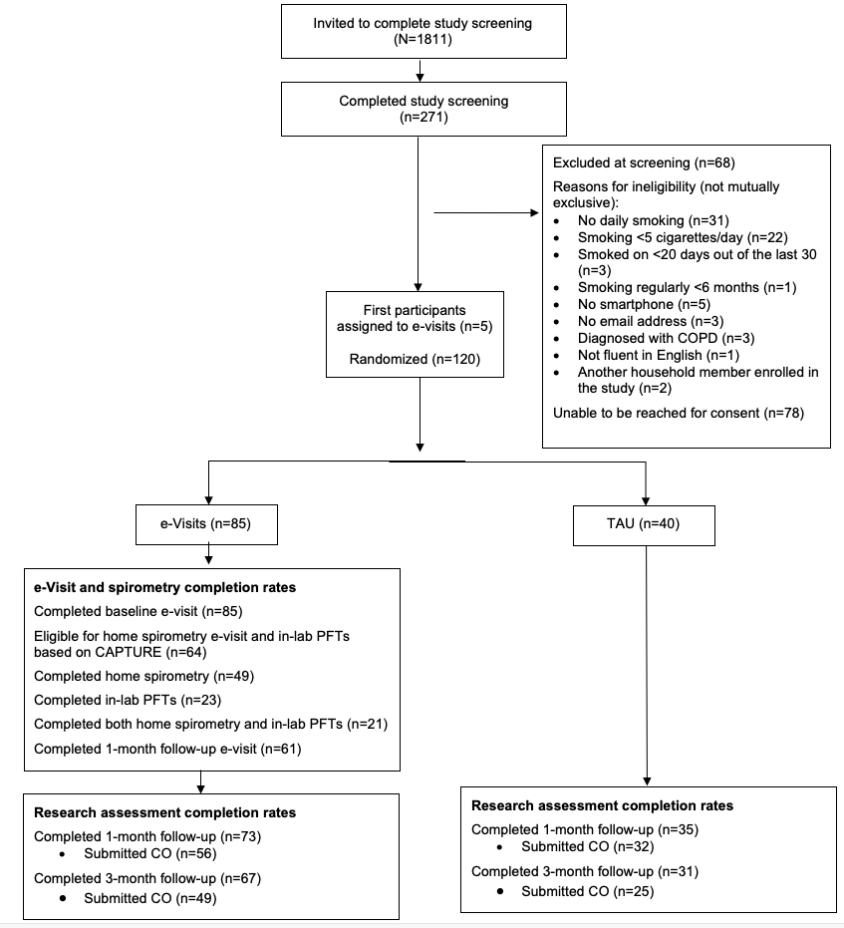
CONSORT flow diagram. CAPTURE: COPD Assessment in Primary Care to Identify Undiagnosed Respiratory Disease and Exacerbation Risk; CO: carbon monoxide; COPD: chronic obstructive pulmonary disease; PFT: peak expiratory flow; TAU: treatment as usual.

Participants were recruited from 13 primary care practices affiliated with MUSC between December 2019 and January 2021. Within our systemwide EHR, Epic, a study recruitment report was generated for all patients meeting the following criteria: (1) aged >40 years; (2) seen at an MUSC primary care practice in the last year; (3) current smoker; (4) no previous diagnosis of COPD (defined as International Classification of diseases, 10th revision codes J44.9, J44.1, J44.0, J43.9, or Z87.9) associated with any prior visit; and (5) access to MyChart, Epic’s patient portal. Via MyChart, 1811 patients meeting initial eligibility criteria were sent an invitation and link to study screening. Study invitations included introductory text highlighting the importance of quitting smoking and then continued with an invitation to participate in a research study to help change smoking behavior. All invitations noted that the study had been discussed with the patient’s primary care provider, who supported the study invitation. Patients were deemed eligible for the study if during the initial screening they met the following additional criteria: (1) current cigarette smoking, defined as smoking 5 cigarettes per day for at least 20 out of the preceding 30 days, for at least the last 6 months; (2) possess a valid email address, checked daily; (3) owner of an iOS or Android-compatible smartphone; and (4) fluent in English (study e-visits were only available in English, thus English fluency was required). In total, 271 patients completed study screening (ie, 15% of those invited), and 203 were deemed eligible following screening (Figure 1).

### Study Procedures

Upon consent, enrolled participants completed baseline assessments via REDCap (version 11.2.1; Vanderbilt University) and then were randomized 2:1 to receive either the e-visits or TAU. The first 5 enrolled participants were all assigned to the e-visit condition to ensure that both smoking cessation and COPD e-visit components were functioning properly. All study participants were invited to complete follow-up research assessments at 1 and 3 months following baseline. To biochemically verify smoking status, participants were asked to submit an expired air carbon monoxide (CO) sample remotely via a smartphone-enabled CO monitor (iCO Smokerlyzer) at both follow-ups. iCO monitors were mailed to all enrolled participants following completion of the baseline visit, and mailings included information regarding how to submit CO readings. Participants were compensated up to US $250 total for all study procedures. Participants randomized to the e-visit condition were not separately compensated for completion of the e-visits or for completion of in-lab PFTs.

### Interventions

#### Smoking Cessation and COPD e-Visits

Participants in this condition were automatically linked to initiate an asynchronous e-visit via MyChart. Smoking cessation components of the e-visit were similar to our team’s prior work [13] but modified to include an additional focus on COPD screening. e-Visit functionality was similar to that of an electronic questionnaire, with participants answering 1 question per screen and then advancing to the next screen. The initial baseline e-visit gathered information about smoking history and motivation to quit, followed by an algorithm to determine the best Food and Drug Administration (FDA)-approved cessation medication (ie, nicotine replacement therapy [NRT], varenicline, and bupropion) to prescribe. This algorithm was based on prior research [17,18] and evidence-based guidelines [7], using branching logic to prioritize the most efficacious medications (ie, varenicline and combination NRT), while tailoring recommendations based on contraindications and participant preference. A medication recommendation was then displayed to the participant, with a personalized rationale, to which the participant could agree or request a different treatment. e-Visit results were then sent to the provider’s electronic in-basket, who reviewed the e-visit, responded to the patient via MyChart with instructions, and e-prescribed (if indicated) medication. All medications were prescribed on label by 3 study physicians and were mailed to the patient at no cost. Responses from providers to participants also included information on the state tobacco Quitline, which participants could contact for additional behavioral support. Participants were subsequently invited to complete a follow-up smoking cessation e-visit 1 month following completion of the baseline session, consistent with the 5th A in the 5 A’s guideline to arrange follow-up [7], at which time participants could request a refill of the medication prescribed at baseline, if needed, or could request a new smoking cessation medication. Results were sent to providers and reviewed in the same manner as the baseline e-visit.

In addition to smoking cessation content, the baseline e-visit included completion of the 5-item COPD Assessment in Primary Care to Identify Undiagnosed Respiratory Disease and Exacerbation Risk (CAPTURE) [19]. CAPTURE assesses the presence or absence of COPD symptoms, risk exposures, and recent history of acute respiratory illness. Responses are summed and scores of 2 or higher suggest a need for diagnostic assessment [5]. Thus, e-visit participants with a CAPTURE score ≥2 were subsequently invited to complete both remote spirometry and in-lab PFTs. For remote spirometry testing, participants were mailed a home spirometer (Vitalograph asma-1) and were sent a link to complete an additional e-visit in which they recorded themselves using the home spirometer. At the beginning of the home spirometry e-visit, participants reviewed an educational video, developed by our team, demonstrating how to use the device, while video recording themselves. Participants submitted 3 breath samples with accompanying videos for evaluation and were asked to enter peak expiratory flow (PEF) and forced expiratory volume in one second (FEV1) values into text boxes within the e-visit. Completed home spirometry e-visits and videos were reviewed by a study physician, who coded them for effort (ie, acceptable, unacceptable, and unable to determine), technique (coded similarly), and whether the participant correctly recorded PEF and FEV1 values from the home spirometer (ie, yes, no, or participant did not show values to the camera). For each participant, the percent predicted PEF was calculated from the highest acceptable PEF recorded, factoring in age, gender, and ethnicity, based on equations with standard values [20,21]. Participants with predicted PEF ≤80% were considered abnormal. Similarly, the highest acceptable FEV1 measurement was categorized as normal (≥80%), moderate (≥50%-<80% predicted), severe (≥30%-<50% predicted), or very severe (<30% predicted). After review, the physician sent a message to the patient via the EHR portal with results (normal or abnormal) along with a recommendation to complete in-lab PFTs previously ordered. These messages also included encouragement related to quitting smoking (eg, “we still recommend that you attempt to quit smoking. Quitting smoking now will help to prevent any further lung damage as well as reduce your risk of heart disease and cancer linked to tobacco use. We are here to continue helping you in those efforts”). No intervention for COPD was provided as part of this study.

All participants eligible for home spirometry testing were also referred for PFTs, regardless of the home testing results, to examine the comparative feasibility of home versus in-lab testing. To further remove barriers to completion of in-lab PFTs, all PFT costs were paid for by the study. Once completed, in-lab PFT results were communicated to the patient with recommendations for follow-up with their primary provider. Chart reviews were completed at 3 months following study enrollment for all study participants to determine whether PFTs were completed and whether the participant was subsequently diagnosed with COPD. PFT appointments for these participants were scheduled per usual practice (ie, a referral was placed by study coordinators, and central scheduling contacted participants to schedule testing).

#### Treatment as Usual (TAU)

TAU was designed to mimic existing standard cessation practices. Research staff provided participants in this condition with information on the state Quitline and a recommendation to contact their primary care provider to schedule a medical visit to discuss quitting smoking. Chart review was also completed at 3 months for these participants to determine whether they completed in-lab PFTs and whether they were diagnosed with COPD.

### Measures

All participants at baseline completed a general assessment of demographics and health history. Primary outcomes for this trial include measures related to (1) e-visit acceptability, feasibility, and treatment metrics; (2) smoking cessation outcomes; and (3) COPD screening outcomes. Unless otherwise noted, participants self-input responses to study outcomes assessments in REDCap.

#### e-Visit Acceptability, Feasibility, and Treatment Metrics

To assess participant perception of the e-visits, during the 1-month research assessment, e-visit participants responded to the following items: (1) I found the e-visit easy to use; (2) I would use an e-visit again in the future; (3) during my e-visit, I felt I could trust my provider with my medical care; (4) I would recommend e-visits to other people; (5) It was as easy for me to state concerns through the e-visit as it would be in an in-person visit; (6) the e-visit was as good as an in-person visit with my doctor; and (7) I have experienced benefits from the e-visit. Response options ranged from strongly disagree to strongly agree.

To examine comparative feasibility of remote versus in-lab PFTs, completion rates for each were captured and compared. Feasibility of remote home spirometry was further assessed through clinician ratings of spirometry effort or technique, as described above. Feasibility of the smoking cessation component of the e-visit was captured via EHR chart reviews, as follows: (1) whether the patient opted for the medication recommended by the e-visit, (2) whether the physician prescribed the medication recommended by the e-visit, (3) whether the participant completed the 1-month follow-up e-visit, and (4) time to complete the 1-month follow-up e-visit.

#### Smoking Cessation Outcomes

All participants at baseline were queried for the number of cigarettes smoked per smoking day, incidence of quit attempts within the last year, and motivation or confidence to quit (0-10 on the visual analogue scale [22]) in the next month. During the 1- and 3-month follow-ups, all participants self-reported the following: (1) number of cigarettes smoked per day over the last 7 days, (2) incidence of 24-hour quit attempts since the prior assessment, and (3) use of an FDA-approved smoking cessation medication since the last assessment. Past-week smoking data allowed for a computed outcome to assess if participants reduced their smoking by at least 50% since baseline. Participants who reported smoking zero cigarettes over the last 7 days were coded as having self-reported 7-day point prevalence abstinence. Self-reported abstinence was biochemically confirmed via CO, using a cutoff point <6 ppm to define abstinence [23].

#### COPD Outcomes

COPD diagnostic status was ascertained for all participants via chart review at 3 months following study enrollment. Additionally, at baseline, all participants completed CAPTURE [19].

### Statistical Analysis Plan

Chi-square and ANOVA analyses were used to determine baseline group differences in participant demographics as well as retention rates over time. Descriptive statistics were used to examine e-visit (for spirometry and smoking cessation) acceptability, feasibility, and treatment metrics. Binary logistic regressions were used to examine differences in cessation outcomes across treatment group, at both 1- and 3-month time points. For cessation outcomes, an intent-to-treat approach was used such that those who did not complete the assessment were coded as not having modified smoking [24].

## Results

### Participant Characteristics

In total, 125 participants were enrolled in the trial (e-visit=85 and TAU=40). The first 5 enrolled participants were all assigned to the e-visit condition, and the remaining 120 were randomized 2:1 to either e-visit or TAU. The first 5 enrolled participants did not significantly differ from those randomized, either in baseline characteristics or follow-up outcomes. There were no significant between-group differences in demographics or smoking history at baseline (Table 1), though participants in the TAU condition reported significantly greater COPD symptoms on CAPTURE (*F*_1,123_=8.11, *P*=.005).

Study retention was generally high across both 1-month (86.4%) and 3-month (78.4%) follow-ups, with no significant differences in retention between treatment groups. Regarding demographic differences between those who completed follow-up assessments and those who did not, White participants were significantly more likely to complete the 1-month follow-up assessment compared to non-White participants (91% completion versus 75%; *χ*^2^_1,125_=5.59; *P*=.02). There were no other significant demographic differences between those who completed follow-up assessments and those who did not, at either 1 or 3 months. Among 1-month respondents, 81.5% (88/108) also provided CO. Among 3-month respondents, 73.5% (72/98) also provided CO.

**Table 1:** Participant demographics.

Characteristics		Full sample (N=125)	e-Visit (n=85)	Treatment as usual (n=40)
Age (years), mean (SD)		53.42 (9.40)	54.12 (9.75)	51.93 (8.52)
**Age (years), n (%)**				
	40-64	109 (87.2)	71 (83.5)	38 (95)
	>65	16 (12.8)	14 (16.5)	2 (5)
Sex (female), n (%)		75 (59.2)	52 (61.2)	22 (55)
**Race, n (%)**				
	White	89 (71.2)	61 (71.8)	28 (70)
	Black	28 (22.4)	18 (21.2)	10 (25)
	Other	8 (6.4)	6 (7.2)	2 (5)
Ethnicity (Hispanic/Latinx), n (%)		4 (3.2)	2 (2.4)	2 (5)
**Education, n (%)**				
	<High school diploma	44 (35.2)	27 (31.8)	17 (42.5)
	>High school diploma	81 (64.8)	58 (68.3)	23 (57.5)
**Annual household income, n (%)**				
	<US $50K	65 (52)	43 (50.6)	22 (55)
	>US $50K	56 (44.8)	39 (45.8)	17 (42.5)
	Not sure or refused to answer	4 (3.2)	3 (3.6)	1 (2.5)
**Health insurance status, n (%)**				
	Total number of participants insured	112 (89.6)	79 (92.9)	33 (82.5)
	Medicaid	15 (12)	10 (11.8)	5 (12.5)
	Medicare	23 (18.4)	17 (20.0)	6 (15)
	Employer-provided insurance	53 (42.4)	37 (43.5)	16 (40)
	Other	21 (16.8)	15 (17.6)	6 (15)
Baseline cigarettes per day, mean (SD)		18.43 (9.79)	18.09 (8.98)	19.15 (11.43)
Quit attempt in the past year (yes), n (%)		73 (58.4)	48 (56.5)	25 (62.5)
Motivation to quit in the next month, mean (SD)		7.53 (2.50)	7.36 (2.56)	7.88 (2.34)
Confidence in quitting in the next month, mean (SD)		5.76 (2.88)	5.64 (2.87)	6.03 (2.92)
Baseline CAPTURE^a^, mean (SD)		3.13 (1.72)	2.84 (1.72)	3.75 (1.56)

^a^CAPTURE: COPD Assessment in Primary Care to Identify Undiagnosed Respiratory Disease and Exacerbation Risk.

### e-Visit Feasibility, Acceptability, and Uptake

**Figure 2:**
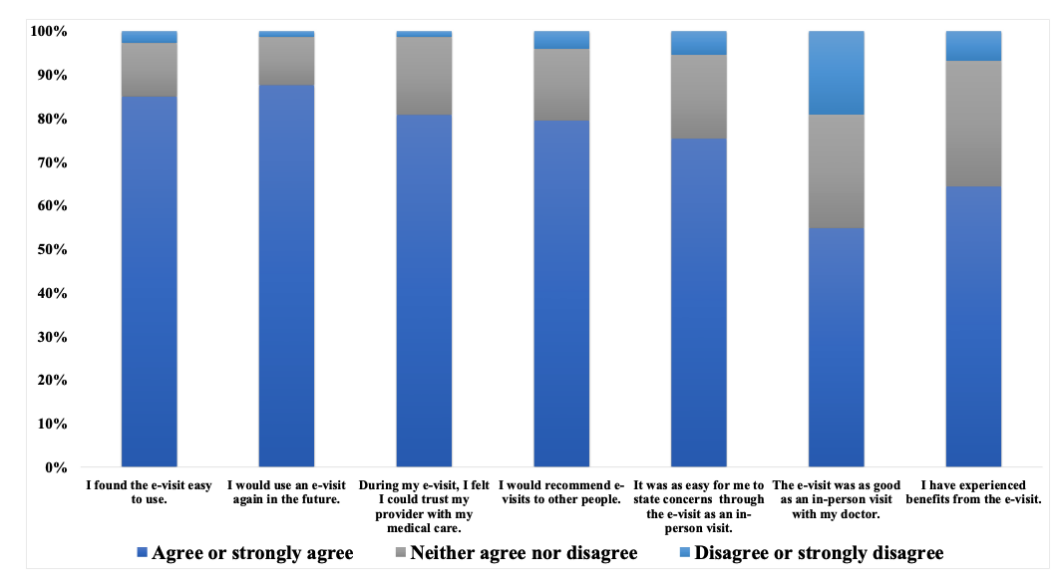
e-Visit feedback.

Participant feedback following completion of the baseline e-visit was generally positive (Figure 2). Of the 85 participants assigned to the e-visit condition, 64 (75.3%) were invited to complete home spirometry and in-lab PFTs because of a CAPTURE score of 2 or higher. Mean CAPTURE score among those eligible for spirometry was 3.7 (SD 1.4). Among those eligible for spirometry, 76.6% (49/64) completed home spirometry. Most of those (37/49; 75.5%) who completed home spirometry had acceptable effort; 79.6% (39/49) had acceptable technique, and 87.8% (43/49) correctly recorded values on at least 1 video. Two-thirds (33/49; 67.3%) of participants who completed home spirometry had at least 1 video with acceptable effort and technique.

**Figure 3:**
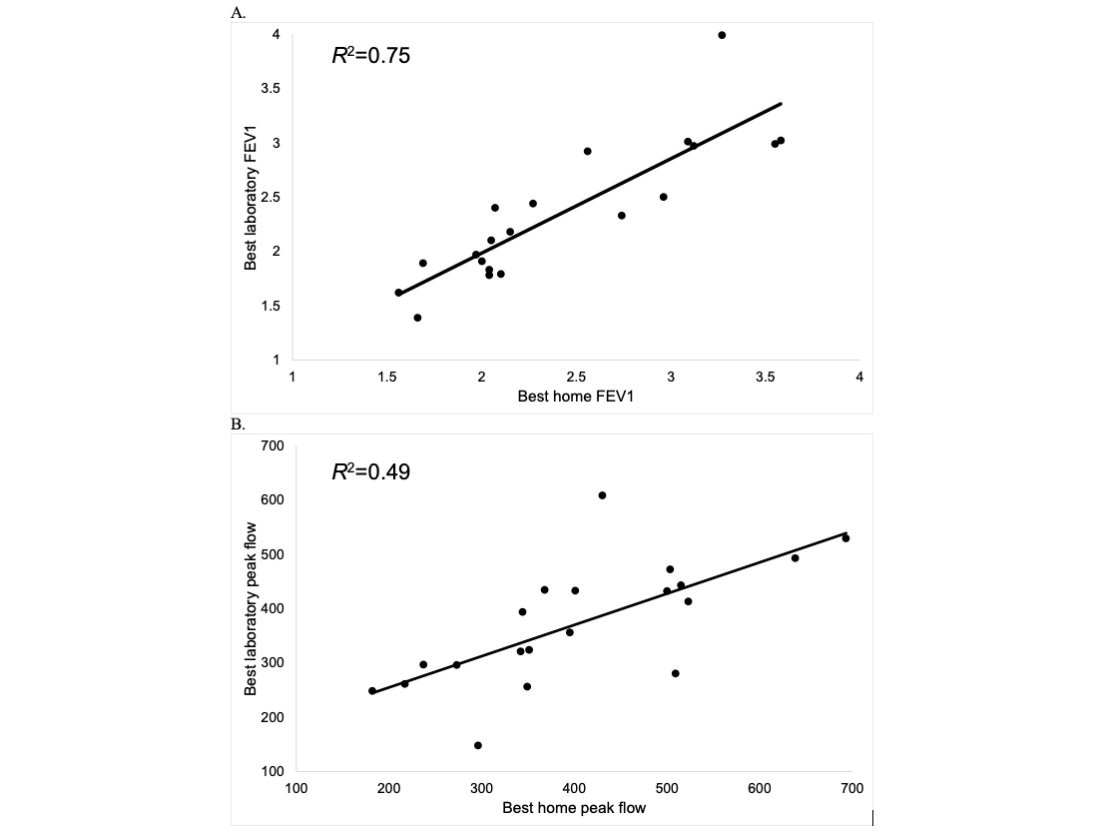
Home vs. In-Lab FEV1 and Peak Flow Among Participants Who Completed Both.

Compared to completion rates for home spirometry, fewer participants in the e-visit condition completed in-lab PFTs (23/64, 35.9% of those eligible). Twenty-one participants completed both home and in-lab PFTs. Among these participants, concordance between home and in-lab PFTs was higher for FEV1 (*R*^2^=0.75) compared to PEF (*R*^2^=0.49; Figure 3). Among the sample completing both home spirometry and in-person PFTs, in-person spirometric diagnoses included normal spirometry (4/21, 19%), Global Initiative for Obstructive Lung Disease undifferentiated obstruction with FEV1 <80% but FEV1/forced vital capacity >0.7 (2/21, 9.5%), probable or confirmed restriction (5/21, 23.8%), mild obstruction (5/21, 23.8%) and moderate obstruction (5/21, 23.8%). No participant was found to have severe obstruction. Using a cutoff of home spirometry PEF <80% predicted, 5 participants who completed both home spirometry and in-person PFTs were considered to have abnormal home spirometry readings. Among these participants, in-person PFTs confirmed restriction (1/5, 20%), mild obstruction (1/5, 20%), moderate obstruction (2/5, 40%), and normal spirometry (1/5, 20%). In the TAU group, only 1 participant completed in-lab PFTs and that participant was subsequently diagnosed with COPD.

### Smoking Cessation, Treatment, and Outcomes

The most common treatment recommendation as a result of the baseline e-visit was varenicline (65/85, 76.5%), followed by NRT patch (8/85, 9.4%), combination NRT (5/85, 5.9%), and NRT lozenge (3/85, 3.53%). Participants (73/85, 86%) and providers (72/85, 85%) agreed with recommendations from the medication algorithm. Three quarters (64/85, 75.3%) of e-visit participants completed the 1-month e-visit, on average within 2.7 (SD 6.9) days after invitation. During this follow-up e-visit, participants most often requested either a prescription for varenicline (20/64, 31.3%), combination NRT (11/64, 17.2%), NRT inhaler (10/64, 15.6%), or NRT gum (6/64, 9.4%), and providers abided by these preferences (63/64, 98.4%).

**Figure 4:**
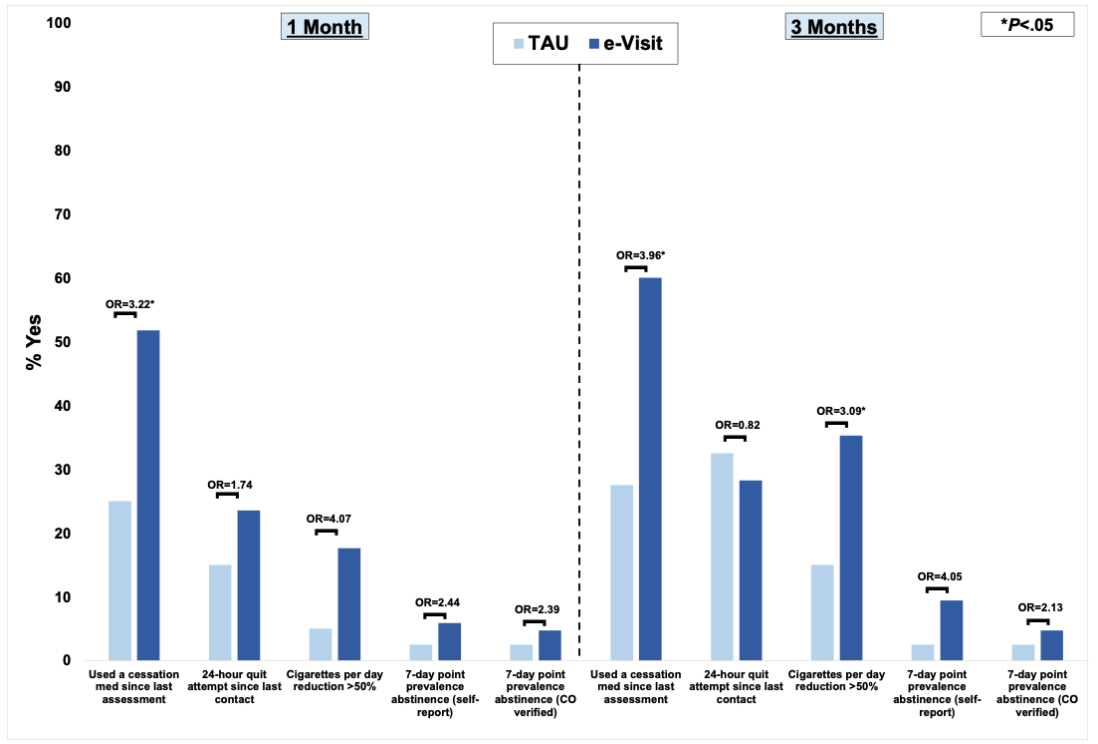
Smoking cessation outcomes. CO: carbon monoxide; OR: odds ratio; TAU: treatment as usual.

In general, smoking cessation outcomes favored the e-visit condition at both 1 and 3 months (Figure 4). At 1 month, all cessation outcomes favored the e-visit condition (ORs 1.6-4.1). At 3 months, all cessation outcomes except for 24-hour quit attempts favored the e-visit condition (ORs 1.1-5.8). Regarding significant effects, as compared to TAU, e-visit participants were 3.2 times more likely to have used a cessation medication at 1 month (95% CI 1.4-7.4; *P*=.006), and 4.0 times more likely to have used a cessation medication at 3 months (95% CI 1.7-9.0; *P*<.001). At 3 months, e-visit participants were 3.1 times more likely to have reduced their cigarettes per day by at least 50% (95% CI 1.2-8.2; *P*=.02), with a similar trend toward significance at 1 month (OR 4.1, 95% CI 0.9-18.8; *P*=.07).

## Discussion

Study results preliminarily indicate feasibility, acceptability, and efficacy of a proactive, asynchronous e-visit for smoking cessation and COPD screening. Metrics of feasibility and acceptability were strong, with 88% (75/85) of participants indicating that they would use an e-visit again in the future. However, only a small majority (47/85, 55%) of e-visit participants reported preferring the e-visit to an in-person visit. Thus, although the e-visit may offer a scalable, feasible method to extend the reach of cessation treatment, it may not be preferred for all patients. Future research should examine which subgroups of patients may be most amenable to receiving cessation treatment via telehealth platforms such as e-visits.

Our study also confirms the feasibility of home spirometry, with promising compliance rates for submission of viable tests. This is consistent with prior research among other populations with obstructive lung diseases such as cystic fibrosis [25,26] and asthma [27]. Thus, home spirometry, completed via an e-visit and reviewed by a provider, may be a useful tool for COPD screening among high-risk smokers. A large body of literature has focused on both the potential utility and drawbacks of population-based screening for COPD, particularly among asymptomatic patients. Current US Preventive Services Task Force guidelines recommend against screening asymptomatic adults for COPD, citing lack of supportive data [28]. However, screening of individuals who self-report unaddressed respiratory symptoms, as implemented in this trial, can increase COPD diagnoses and facilitate treatment initiation [10-12]. Although a tool such as CAPTURE has broad reach and may help to identify those who are symptomatic, pairing CAPTURE with home-based spirometry could help further identify the subset of patients who should be strongly encouraged to complete diagnostic PFTs.

This study was not designed specifically to examine the validity of home spirometry; nevertheless, comparing home versus in-lab results for the small subset of participants who completed both suggests there are opportunities to improve validity. To maximize scalability of the e-visits, we opted to minimize the amount of training provided to participants. However, prior home spirometry trials have had success with incorporating synchronous coaching via video calls [29]. This approach may help improve the validity of home spirometry among adult smokers at risk for COPD, though it would limit scalability. In the future, it will be important to determine the appropriate amount of training needed for participants to submit valid samples and how best to embed this training in primary care.

To our knowledge, this is the first study to evaluate the CAPTURE, which was developed to be used in a broad population of primary care patients, in a population of smokers. We were surprised to see that 82.4% (103/125) of smokers in our full sample had a score of 2 or higher at baseline. Because smokers typically develop COPD after 10 pack years at a prevalence <20% [30], the CAPTURE instrument should be reevaluated among active smokers, as sensitivity may be too high. This is likely due to scoring 1 point for living or working in a place with smoke or secondhand smoke.

Our results further substantiate the potential of e-visits to promote smoking cessation. Results generally echoed those of our prior trial [13], supporting the efficacy of the e-visit approach. Whereas our prior trial did not provide free medications, the current study provided prescribed medications free of charge. Comparing intent-to-treat results across studies, the provision of free medication appears to have slightly increased the use of cessation medications (at 1 month: 44.1% vs 51.8%; at 3 months: 41.2% vs 60%). Thus, where possible, pairing the proactive e-visit with free medication may increase evidence-based cessation treatment uptake.

Results of this study should be interpreted with limitations in mind. The trial was largely conducted in the midst of the COVID-19 pandemic, which may have impacted rates of trial enrollment, engagement with the e-visits, and completion rates for in-lab PFTs. However, it is important to note that at MUSC, pulmonary function testing continued throughout the pandemic, thus all participants eligible for in-lab PFTs had the option to complete them. Future evaluation of proactive e-visits for COPD screening and smoking cessation outside the context of the COVID-19 pandemic will be important to determine whether e-visit acceptability and feasibility change as a function of the pandemic waning. Regarding generalizability, the Vitalograph asma-1 was used for remote home spirometry completion. Feasibility and validity results may not generalize to other remote monitors. Moreover, study inclusion criteria, including smartphone ownership and regular email use, may limit generalizability of results. Proactive study invitations were sent via MyChart, which may also decrease results’ generalizability. However, this decision was made because the study e-visits were delivered via the MyChart patient portal. Given the preliminary nature of this trial and focus on feasibility and acceptability, resources to support deployment of both remote spirometry and in-lab PFTs were not comprehensively assessed. However, future cost-effectiveness analyses could help determine whether potential benefits of the e-visit approach are cost-effective or cost-saving at the health care system level. Finally, the completed e-visits were reviewed by study physicians. Implementation of the e-visits within routine clinical practice and with non–study-affiliated providers remains unclear but is an important avenue for future research.

In sum, a proactive, asynchronous e-visit for smoking cessation treatment and COPD screening may offer a feasible, efficacious approach for broad intervention within primary care. If validity of home spirometry can be improved over time, the e-visit platform may help not only promote uptake of evidence-based smoking cessation treatment but also provide an early screening mechanism to identify smokers with COPD or other important lung diseases.

## Appendix

Multimedia Appendix 1 CONSORT-eHEALTH checklist (V 1.6.1).

## Abbreviations

CAPTURE: COPD Assessment in Primary Care to Identify Undiagnosed Respiratory Disease and Exacerbation Risk
CO: carbon monoxide
COPD: chronic obstructive pulmonary disease
EHR: electronic health record
FDA: Food and Drug Administration
FEV1: forced expiratory volume in one second
MUSC: Medical University of South Carolina
OR: odds ratio
PEF: peak expiratory flow
PFT: Pulmonary Function Test
TAU: treatment as usual
